# Case of Labor, with Spontaneous Evolutions, &c.

**Published:** 1856-04

**Authors:** A. M. Carpenter


### A CASE OF LABOR, WITH SPONTANEOUS EVOLUTIONS, &c.,

BY A. M. CARPENTER, M. D.

On the night of the 12th of Sept. 1855, I was summoned to see Mrs. C., residing on Johnson street, æt. 24, then in labor with her second child. After making the usual enquiries of the nurse, I ascertained that the waters had been discharged nine hours since, and that the patient had experienced but slight pains during the day, not at any period warranting her in seeking the recumbent position up to the present. Upon examination as to her general condition and finding her quite comfortable, I proceeded to a vaginal exploration. The soft parts were well dilated, the vaginal track moist and of proper temperature; upon a further prosecution, my index reached the Os, and found it in an irregular, unnatural condition, the anterior lip projecting to the extent of half an inch, almost completely obliterating its fellow; the inferior lip tense and contracted, partaking much of the feel of a cicatrix. The Os not being sufficiently patulous to reveal the true nature of the presenting part, led me at once to administer a nauseant, the effect of which was, in a short time, well observed, thereby enabling me to more accurately fix the diagnosis. My finger was re-introduced, and after slightly elevating the superior lip, then quite tractable, came in contact with what I supposed to be the buttock, and upon a little manipulation, passed into the anus, the withdrawal of which characterized with precision, the true presentation, that of the pelvic variety, occupying the right dorso iliac position, the posterior plane of the fœtus looking towards the right side of the mother. The premature rupture of the bag of waters and the enfeebled contractile efforts of the uterine fibre, almost confirmed the suspicion that the fœtus had materially and fatally suffered from the prolonged adaptation and compression of the uterine parietes.

I accordingly auscultated the abdomen, though no fœtal click or placental sounds were elicited, of course, farther tending to verify the surmise as to its death. I for some time patiently awaited a recurrence of the pains, though in vain. In the meantime the patient become very anxious and irrascible, wishing her labor completed, appealing, not unfrequently, for aid, and growing less and less confident that her delivery would ever be effected, unless by immediate interference upon the part of the Accoucheur. All and every attempt to encourage her and to assuage her peculiarly mean temper were utterly futile, and only exasperated instead of palliating her. I at length, after the natural resources seemed unwilling to participate in the contest, as an auxiliary, commenced using gentle friction over the lower belly, which was persevered in for a time; the uterus, however, failed in any essential degree to respond to it. The patient by this time had lost all hope, and become wholly intractable, rejecting any and every counsel from myself, her family and friends, and expressed a desire to get up, which she was allowed, and after pacing her apartment a few times, revelling in a sort of wild delirium, returned to her bed. The parturient powers having had sufficient time to take on their characteristic action, in the expulsive drama, and refusing to deal herself a hand. I then deemed it wise to inform Madam Nature, that a system of action foreign to her, should be introduced whereby she would be aroused from her dormancy, and made familiar with an interposing agent, which defies all powers and antagonizes all her best dealt efforts at resistance, namely the Secale Cornutum, which was administered in the ordinary dose, and its action patiently awaited. In a brief space the uterus began its response, its action was tardy for two or three hours, while the fœtus made but scarce appreciable progress, the patient maintaining her strength remarkably well. Near about 5 o'clock, the medicament was repeated, which hastily awakened the uterus to its sense of incumbency, and although the contractions were as rapid and vigorous as I would desire them, the advance of the occult body was *but* perceptible. About 7 o'clock, I took leave for breakfast, and when ascending the stairway to my office, I was hailed by the husband, saying, Mr. Doctor, something has burst. I, of course, readily imagined a rent in the uterus. My steps, however, were hastily retraced, while my mind revolved the meeting of a terrible catastrophe; the patient either dead or dying. Upon examination, an amazingly agreeable fact was unfolded. The first obstacle my hand encountered was the right arm protruded into the external world. I thought it a happy evolution, both for the mother and myself, when contrasting it with my very disagreeable expectations before I reached the house. The fœtus then maintained the first position of the right shoulder, in which the head was unusually flexed upon the sternal bone, not seeming, as it were, to occupy the left Iliac Fossa, as it is ordinarily wont to do, in the first position of either shoulder, and accounted for by the amassed situation which it must almost necessarily attain, under the circumstantial force, brought to bear directly upon it; while as usually, the breech rested in the right Iliac Fossa—the back or posterior plane looking forward, and the breast or anterior plane looking directly backward.

The idea to turn and deliver was the next step to be taken, as I granted it one of the very greatest physical impossibilities for the inherent resources to surmount, and in the first intermission, seized the fore arm, flexed it and attempted to return it, but was foiled by the most vehement and quick succeeding pains I have ever witnessed, providentially or otherwise, if you please. A short respite was allowed, and in the interim, as fast as thought, I again grasped the arm and suceeded in replacing it, almost simultaneously pressing upon the shoulder in an upward and backward tendency, which, as I thought, would and did exert a tendency (by continuing the pressure) to bring down the head, and, in truth, such was the case in the subsequent pain, which so completely amassed and jammed the head and thorax in the basin of the Pelvis and against the then attenuated Perinæum, that I greatly feared its rending asunder; the shoulder or counter pressure was persevered in during each pain until I was enabled to apply my index finger in the form of a blunt hook to the cervix of the fœtus, when by gentle traction, in co-operation with the contraction, the head and shoulders were expelled en masse, or if you like, the head flexed upon the chest, while the remainder of the trunk and limbs were issued by the following pain, and what is more remarkable to observe and equally gratifying, without the least imaginable damage to the soft parts of the mother. The child was still born, weighing accurately 13¼ pounds, and exhibited appearances of having been dead several days.

The placenta I discovered to be morbidly adherent, which I

detached piece by piece, being followed by slight hæmorrhage; tonic contraction, however, was almost immediately set up by the ordinary manipulations, and the hæmorrhage ceased. The patient being pretty well exhausted, was allowed a little wine. She soon sank into a pleasant slumber, which she enjoyed for two hours or more, and upon my visit eight hours hence, found her quite comfortable, free from pain and tenderness, from which time she rapidly convalesced, without an untoward symptom.

REMARKS.—Hoping that the brief assembly of phenomena in this case may prove of utility to some member of the profession, I submit it for your consideration, not however from its novelty or unfrequent occurrence, but from the clear exemplification of the abundant aid that may be looked for when the intrinsic forces of nature are brought into requisition; nor shall I in any sense be comprehended as urging or recommending your interposition in expectancy of such a result, lest there be obstacles of so great magnitude as to preclude all possibility of a remedial or amendable agent; even then it should be submitted to the wisest and best pointed thought. The palpability of the case just considered, should, in no large degree, warrant the confiding of a similar one to the unabetted powers of nature, however much we may admire the vastness and excellence of her resources, and however manifoldly we may appreciate her untiring efforts, which she not unfrequently exerts when foreign aid proves utterly futile.

The points in this case will be seen to be the following, viz: Premature discharge of the liquor Amnii, adaptation of the Uterus to the inequalities of the Fœtus, ridigity of the Os, breech presentation, spontaneous evolution and delivery by the left shoulder.

